# Anterior Slope–Modifying Osteotomies Alter the Length Change Behavior of the Superficial Medial Collateral Ligament: A Biomechanical Study

**DOI:** 10.1177/03635465241280985

**Published:** 2024-10-06

**Authors:** Christian Peez, Carla Ottens, Adrian Deichsel, Michael J. Raschke, Thorben Briese, Elmar Herbst, James R. Robinson, Christoph Kittl

**Affiliations:** † Department of Trauma, Hand and Reconstructive Surgery, University Hospital Münster, Münster, Germany; ‡ Knee Specialists, Bristol, United Kingdom; Investigation performed at University Hospital Münster, Münster, Germany

**Keywords:** superficial medial collateral ligament, sMCL, length change pattern, posterior tibial slope, slope modification, biomechanics

## Abstract

**Background:**

Increased tibial slope has been shown to lead to higher rates of anterior cruciate ligament graft failure. A slope-decreasing osteotomy can reduce in situ anterior cruciate ligament force and may mitigate this risk. However, how this procedure may affect the length change behavior of the medial ligamentous structures is unknown.

**Purpose/Hypothesis:**

The purpose of this study was to examine the effect of anterior slope–modifying osteotomies on the medial ligamentous structures. It was hypothesized that (1) decreasing the tibial slope would lead to shortening of the superficial medial collateral ligament (sMCL), (2) while the fibers of the posterior oblique ligament (POL) would be unaffected.

**Study Design:**

Descriptive laboratory study.

**Methods:**

Eight fresh-frozen cadaveric knee specimens underwent anatomic dissection to precisely identify the medial ligamentous structures. The knees were mounted in a custom-made kinematics rig with the quadriceps muscle and iliotibial tract loaded. An anterior slope–modifying osteotomy was performed and fixed using an external fixator, which allowed modification of the wedge height between −15 and +10 mm in 5-mm increments. Threads were mounted between pins positioned at the anterior, middle, and posterior parts of the tibial and femoral attachments of the sMCL and POL. For different tibial slope modifications, length changes between the tibiofemoral pin combinations were recorded using a rotary encoder as the knee was flexed between 0° and 120°.

**Results:**

All sMCL fiber regions shortened with slope reduction (*P* < .001) and lengthened with slope increase (*P* < .001), with the anterior sMCL fibers more affected than the posterior sMCL fibers. A 15-mm anterior closing-wedge high tibial osteotomy (ACWHTO) resulted in a 6.9% ± 3.0% decrease in the length of the anterior sMCL fibers compared with a 3.6% ± 2.3% decrease for the posterior sMCL fibers. A 10-mm anterior opening-wedge high tibial osteotomy (AOWHTO) increased anterior sMCL fiber length by 5.9% ± 2.3% and posterior sMCL fiber length by 1.6% ± 1.0%. The POL fibers were not significantly affected by a slope-modifying osteotomy.

**Conclusion:**

Tibial slope–modifying osteotomies changed the length change pattern of the sMCL such that an AOWHTO increased whereas an ACWHTO decreased the sMCL strain. This effect was most pronounced for the anterior fibers of the sMCL. The length change pattern of the POL remained unaffected by slope-modifying osteotomy.

**Clinical Relevance:**

Surgeons should be aware that anterior tibial slope–modifying osteotomies affect the biomechanics of the sMCL. After an extensive ACWHTO, patients may develop a medial or anteromedial instability, while an AOWHTO may overconstrain the medial compartment.

Despite advances in anterior cruciate ligament (ACL) reconstruction (ACLR), rates of graft failure and residual pivot-shift laxity range from 5% to 20%.^37,38,41,51,53^ Graft selection, tunnel position, and rotatory knee instability may all influence ACLR outcomes, but high posterior tibial slope is increasingly recognized as an important risk for graft failure.^3,11,12,16,17,35,42,50^ Regardless of the measuring technique, increased tibial slope has been shown to increase the grade of the pivot-shift test, anterior tibial translation, and thus in situ graft forces.^1,9,10,14,19,26,47^ Therefore, a tibial slope ≥12° has been demonstrated to be a risk factor for recurrent instability after ACLR.^20,42,50^ Although recent biomechanical studies have shown that tibial slope–decreasing osteotomy can reduce loads on the ACL graft, short-term results for combined slope-reducing osteotomy and ACLR in patients with a high native posterior tibial slope appear promising for both primary and revision ACL surgery.^3,10,12,25,44,45^

However, the fibers of the superficial medial collateral ligament (sMCL) cross the plane of anterior slope–modifying high tibial osteotomy and there is thus a potential to alter ligament length behavior.^5,21^ Slope reduction may result in a shorter distance between femoral and tibial sMCL attachments, thus slackening the sMCL. Persistent medial laxity has been shown to be a risk for ACL graft failure.^4,31^ The sMCL, especially the anterior fibers, is an important restraint to anteromedial rotatory instability.^22,52^ Conversely, increasing tibial effect may have the opposite effect and tighten the sMCL.

Therefore, the purpose of this study was to examine the length change behavior of the sMCL and posterior oblique ligament (POL) after a slope-modifying high tibial osteotomy. It was hypothesized that (1) a slope-decreasing osteotomy would result in a reduction in the distance between the femoral and tibial attachments of the sMCL, whereas (2) a slope-increasing osteotomy would increase the distance. It was further hypothesized that the fibers of the POL would be unaffected by a slope-modifying high tibial osteotomy because of their more posterior tibial attachments.

## Methods

Eight fresh-frozen cadaveric knee specimens (mean age, 74 ± 10.3 years; 4 female, 4 male) with no history of previous ligamentous or bony injury were obtained from an international tissue bank (MedCure). The knee specimens were dissected and biomechanically tested with the permission of the Institutional Ethics Committee (File No. 2022-198-f-S).

### Specimen Preparation

The cadaveric knee specimens were stored at −20°C and thawed at room temperature for 24 hours before anatomic dissection. After cutting the femur and tibia 200 mm proximal and distal to the joint line, respectively, the fibula was cut and secured to the tibia in its anatomic position by a tri-cortical position screw. An intramedullary stainless steel rod (12.0-mm diameter) was cemented into the femoral shaft using polymethylmethacrylate. Two bicortical screws were used for additional rotational stability. After removal of the skin and subcutaneous tissue, the fascia of the medial compartment was dissected to carefully expose the femoral and tibial attachment sites of the sMCL and POL.^39,49^ Similar to previous studies, the iliotibial tract and quadriceps muscle were divided into 6 different anatomic parts: iliotibial tract, vastus lateralis longus, vastus lateralis obliquus, rectus femoris, vastus medialis obliquus, and vastus medialis longus.^27,28,46^ Braided composite sutures (No. 2 FiberWire; Arthrex), used as loading cables, were sutured to the musculotendinous junction of each muscle part with a Krackow locking stitch technique to prevent slippage.^34^

In each specimen, an anterior slope–modifying high tibial osteotomy was performed using the technique described by Hess and Petersen.^21^ After exposure of the tibial tuberosity, the osteotomy plane was defined using four 2.0-mm Kirschner wires (K-wires), placed under radiographic C-arm control, with the tips converging on the posterior cruciate ligament tibial attachment. The anterior entry points of the 2 K-wires defining the proximal osteotomy plane were just below the most distal fibers of the patellar tendon. The distal osteotomy plane was defined by 2 K-wires placed 15 mm below the proximal wires. Two Steinmann pins were then placed under radiographic control 15 mm distal to the joint line and parallel to the articular surface. A further Steinmann pin was placed into the tibial shaft distal to the marked osteotomy planes, and an external fixator was mounted between the pins. This allowed the tibial slope to be incrementally adjusted later.

**Figure 1.**
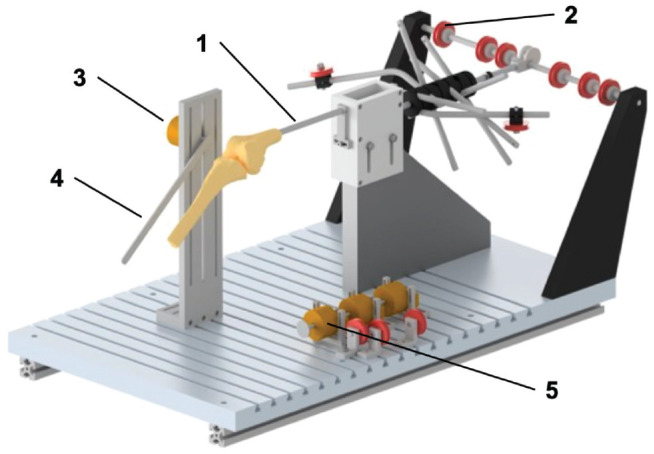
Custom-made kinematics rig adapted from Ghosh et al^18^ and modified by Kittl et al.^28^ The knee specimens were secured to the rig by a femoral intramedullary rod (1). Using a pulley system (2) and hanging weights, the iliotibial tract and quadriceps muscle components were loaded with 205 N, which resulted in a passive extension of the knee joint. Knee flexion was captured using a rotary encoder (3), which was attached to the tibial shaft via a metal bar and a K-wire (4). During manual knee flexion, a second rotary encoder (5) recorded the distance between 2 tibiofemoral attachment combinations.

Each specimen was then mounted in a custom-made kinematics rig (Figure 1). The femoral intramedullary rod was secured so that the posterior femoral condyle axis was parallel to the base of the rig. The iliotibial tract and quadriceps muscle divisions were loaded in total with 205 N according to their physiologic cross-sectional areas and fiber orientation using hanging weights via a pulley system.^13,28,46^ This quadriceps tension extended the knee, which could then be manually flexed from 0° to 120°. This setup has been previously shown to have a high retest reliability.^28,46^

**Figure 2.**
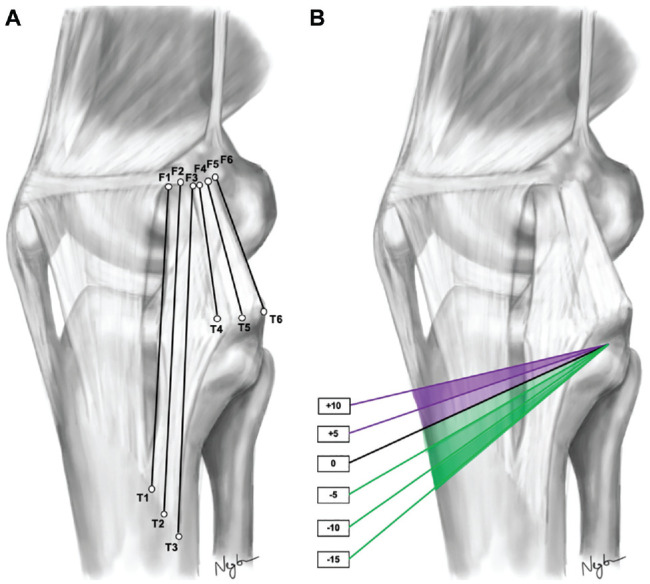
(A) Representative fibers of the superficial medial collateral ligament (sMCL) and posterior oblique ligament (POL) tested. Anterior fibers of the sMCL (T1-F1), middle fibers of the sMCL (T2-F2), posterior fibers of the sMCL (T3-F3), anterior fibers of the POL (T4-F4), middle fibers of the POL (T5-F5), posterior fibers of the POL (T6-F6). (B) In each knee specimen, an anterior opening-wedge high tibial osteotomy was performed with a wedge height of +5 mm and slope increase of +10 mm (purple wedges), which was followed by an anterior closing-wedge high tibial osteotomy with a wedge height of −5 mm and slope reduction of −15 mm (green wedges).

After careful differentiation between the sMCL and POL, the tibial and femoral attachment sites of the anterior, middle, and posterior fibers of the sMCL and POL were marked using small pins (Figure 2, Table 1). For the sMCL, the most anterior and posterior fibers were marked at the anterior and posterior edges of their distal tibial attachment, while the middle fibers of the sMCL were marked equidistantly between them. As previously described, the femoral attachment of the sMCL was consistently found to envelop the medial epicondyle after careful anatomic dissection.^28,32^ Accordingly, the anterior and posterior fibers of the sMCL were marked at the anterior and posterior edges of the medial epicondyle, respectively, while the middle fibers of sMCL were marked at the proximal edge of the medial epicondyle. The most anterior fibers of the superficial arm of the POL were traced to their tibial and femoral attachment and marked with pins. The most posterior fibers of the capsular arm of the POL were marked at their tibial and femoral attachment site in a similar manner. The central arm of the POL was defined as the equidistance between the anterior and posterior fibers of the POL, which were traced to the tibial and femoral attachment and marked with pins.

**Table 1:** Position of the Tibial and Femoral Pins and the Corresponding Tibiofemoral Combination of Each Anatomic Part of the sMCL and POL^*a*^

Tibiofemoral Combination	Position of the Tibial Pin (T1-T6)	Position of the Femoral Pin (F1-F6)
Anterior fibers of the sMCL (T1-F1)	Anterior distal tibial attachment of the sMCL	Anterior edge of the ME
Middle fibers of the sMCL (T2-F2)	Middle distal tibial attachment of the sMCL	Superior border of the ME
Posterior fibers of the sMCL (T3-F3)	Posterior distal tibial attachment of the sMCL	Posterior border of the ME
Anterior fibers of the POL (T4-F4)	Anterior border of the tibial POL attachment	Anterior border of the femoral POL attachment
Medium fibers of the POL (T5-F5)	Middle portion of the tibial POL attachment	Middle portion of the femoral POL attachment
Posterior fibers of the POL (T6-F6)	Posterior portion of the tibial POL attachment	Posterior border of the femoral POL attachment

a ME, medial epicondyle; POL, posterior oblique ligament; sMCL, superficial medial collateral ligament.

### Measurements

Changes in the degree of knee flexion and the length of the sMCL and POL were captured as previously described.^28^ Knee flexion was measured using an optical rotary incremental encoder (PRID 58H8; Opkon), attached to the rig, with its center aligned to the flexion-extension axis. A 2.4-mm K-wire was drilled through the tibial shaft and attached to a metal bar connected to the rotating part of the rotary encoder, so that the angle of knee flexion could be measured to an accuracy of ±0.08°. To investigate sMCL and POL fiber length change behavior, a braided composite suture was tied to a tibial pin, guided to its corresponding femoral pin, and led to a 100 mm–circumference custom-made rubber-edged measuring wheel, attached to a second optical rotary incremental encoder. The suture was held taut with a 0.3-N weight so that friction between the braided suture and the rubber-edged wheel resulted in rotation. The signals from the rotary encoder were recorded using a microcontroller and a computer interface. Signals were converted for each tested combination into knee flexion (degrees; rotary encoder 1) and length changes (millimeters; rotary encoder 2). The accuracy of the rotary encoder was ±0.08°, which allowed length changes to be calculated with an accuracy of ±0.02 mm.

For each testing condition, fiber length was measured between 0° and 120° in 10° increments. This allowed the length change patterns of the sMCL and POL fibers to be plotted. The absolute length of each tested tibiofemoral attachment combination was measured at full extension with a digital caliper (accuracy, ±0.01 mm) to calculate the fiber region–specific strain [(length change/absolute length at 0° of flexion) × 100%]. Fiber isometry was determined by the total strain range (TSR).^23^ The TSR was calculated by subtracting the minimum strain from the maximum strain. High TSR values reflected nonisometry, whereas low values of TSR indicated near isometry.^28^

### Slope Modification

**Figure 3.**
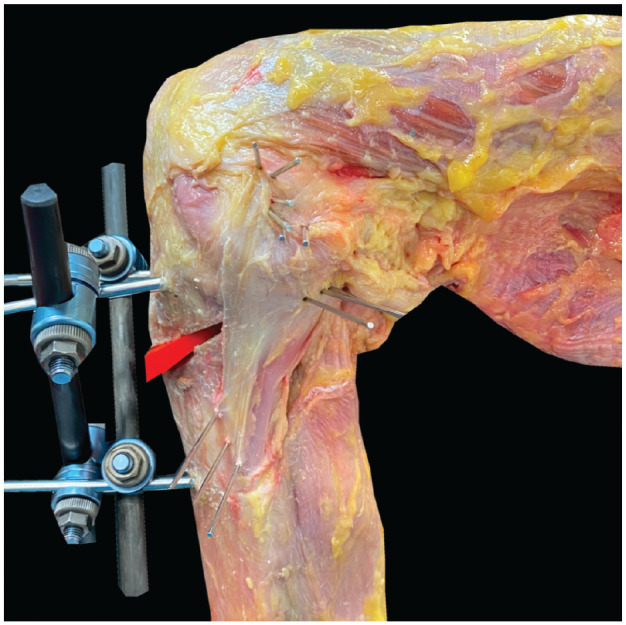
A right knee at 90° of flexion showing the external fixator used to open and close the tibial slope–changing osteotomy. The osteotomy gap was filled with a 3D-printed wedge to improve the stability of the construct during knee flexion. Pins are shown marking the anterior, middle and posterior parts of the tibial and femoral attachment sites of the superficial medial collateral ligament and posterior oblique ligament.

After completing the fiber length change measurements of the native knee joint, a proximal osteotomy was made in the plane of the 2 proximal K-wires using an oscillating saw. The external fixator, attached to the proximal and distal tibial Steinman pins, was then used to open the osteotomy by 5 mm at the anterior tibial cortex. The external fixator was secured, and to improve the stability of the anterior opening-wedge high tibial osteotomy (AOWHTO), the osteotomy gap was filled with a 3D-printed wedge (Figure 3). The measurements of sMCL and POL fiber length changes at each knee flexion angle were repeated. The external fixator was then loosened and the osteotomy opened to 10 mm at the anterior tibial cortex. After insertion of a 10-mm wedge, the external fixator was again tightened and measurements repeated.

To create an anterior closing-wedge high tibial osteotomy (ACWHTO), a second osteotomy was made in the plane of the distal tibial K-wires, allowing a 15-mm, apex-posterior wedge of bone to be removed. The osteotomy gap was closed by 5 mm, 10 mm, and 15 mm. In each position, the external fixator was secured and the osteotomy stabilized with a 3D-printed wedge filling the remaining osteotomy gap. After each adjustment of the tibial slope, the measurements of the sMCL and POL length changes were then repeated.

### Statistical Analysis

A priori power analysis using G*Power (University of Duesseldorf, Germany) showed that a sample size of 8 specimens would lead to 80% power to detect a difference of 1% strain and 3% TSR at a beta level >0.8 based on the standard deviations of previous studies.^28,54^

Statistical analysis was performed using Prism (Version 9; GraphPad Software). The data normality was tested and proved by using the Shapiro-Wilk test. For each fiber region, 2-way repeated-measures analyses of variance with Geisser-Greenhouse correction were performed to compare the length changes (dependent variable) across the slope modifications and different knee flexion angles. Further 2-way repeated-measures analyses of variance with Geisser-Greenhouse correction were performed to compare the TSR of each fiber region across the tibial slope modification. Post hoc Dunnett correction was used to control for multiple comparisons. Statistical significance was set to *P* < .05. The data are presented as mean ± standard deviation.

## Results

### Native sMCL

**Figure 4.**
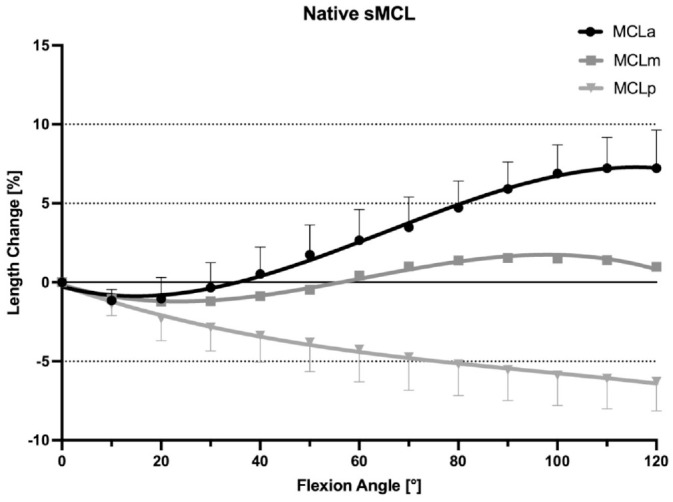
Length change pattern of the 3 fiber regions of the native superficial medial collateral ligament (sMCL), presented as means ± pooled standard deviations. The anterior fibers lengthened during flexion, whereas the posterior fibers shortened with knee flexion. MCLa, anterior fibers of the sMCL; MCLm, middle fibers of the sMCL; MCLp, posterior fibers of the sMCL.

With the knee at 0° of flexion, the mean distances between the pins demarcating the anterior, middle, and posterior fibers of the sMCL were 99.6 ± 8.6 mm, 109.46 ± 9.03 mm, and 11.6 ± 9.2 mm, respectively. During knee flexion-extension, the different fiber regions of the sMCL fibers demonstrated a reciprocal length change pattern. With progressive knee flexion, the distance between the anterior fiber tibiofemoral attachments increased and thus the fibers tightened, while the distance between the attachment sites of the posterior fibers decreased and thus these fibers slackened (*P* < .01). With knee flexion from 0° to 10°, the anterior fibers of the sMCL showed a small but significant initial shortening (–1.1% ± 0.7%; *P* < .05), but then lengthened with increasing flexion to 110° (7.2% ± 1.9%; *P* < .05). Conversely, the posterior fibers of the sMCL continuously shortened between 0° and 120° of knee flexion (–6.3% ± 1.8%; *P* < .05 from 0° to 30°, *P* < .01 from 30° to 120°). The middle fibers of the sMCL demonstrated a sine wave behavior. There was minimal shortening (–1.2% ± 0.8%; *P* < .05) between 0° and 20°, and then lengthening between 20° to 90° of knee flexion (1.5% ± 0.9%; *P* < .05 from 20° to 60°, *P* < .01 from 60° to 90°). There was no significant change in length between 90° and 110° of flexion (1.4% ± 0.9%) (Figure 4). The middle fibers of the sMCL were the most isometric (TSR: 3.4% ± 0.9%), compared with the anterior fibers (TSR: 9.2% ± 3.1%; *P* < .001) and the posterior fibers (TSR: 6.3% ± 1.8%; *P* < .001), which were the least isometric (Table 2).

**Table 2:** Total Strain Range of the sMCL and POL Fiber Regions for Different Anterior Tibial Slope–Modifying Osteotomies^*a*^

Slope, mm	MCLa	MCLm	MCLp	POLa	POLm	POLp
+10	8.9 ± 3.5	3.3 ± 1.0	7.0 ± 2.4	31.7 ± 7.8	42.1 ± 6.2	55.1 ± 5.4
+5	9.2 ± 3.5	3.2 ± 1.4	6.8 ± 2.5	34.4 ± 9.7	46.3 ± 9.4	57.1 ± 9.9
0	9.2 ± 3.0	3.4 ± 0.9	6.2 ± 1.8	29.1 ± 6.7	42.1 ± 6.4	54.4 ± 9.6
−5	9.1 ± 1.9	3.7 ± 1.8	6.8 ± 2.7	33.1 ± 4.6	43.6 ± 6.6	56.7 ± 9.0
−10	8.9 ± 2.4	3.7 ± 1.8	7.1 ± 2.5	32.2 ± 5.4	45.7 ± 5.0	59.1 ± 8.7
−15	9.3 ± 2.6	3.7 ± 1.8	7.1 ± 2.3	32.0 ± 4.4	47.9 ± 4.0	60.4 ± 7.2

a Data are presented as mean ± SD in percentage. Tibial slope modification had no effect on the isometric behavior of the fibers. MCLa, anterior fibers of the sMCL; MCLm, middle fibers of the sMCL; MCLp, posterior fibers of the sMCL; POL, posterior oblique ligament; POLa, anterior fibers of the POL; POLm, middle fibers of the POL; POLp, posterior fibers of the POL; sMCL, superficial medial collateral ligament.

### Native POL

**Figure 5.**
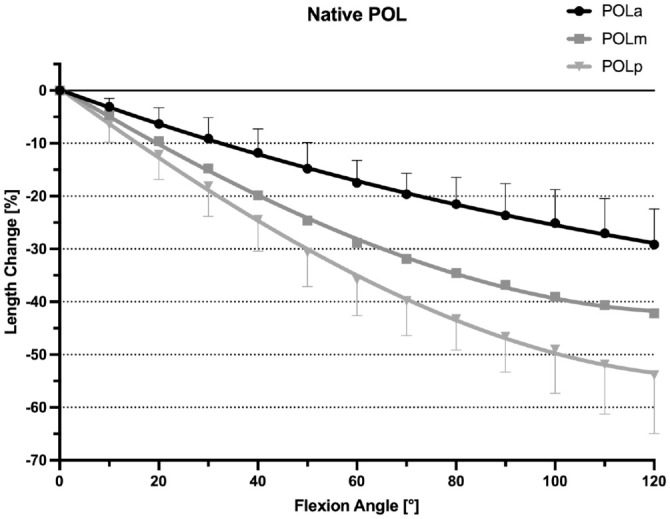
Length change pattern of the 3 fiber regions of the native posterior oblique ligament (POL), presented as means ± pooled standard deviations. All 3 fiber regions showed a uniform shortening with knee flexion. POLa, anterior fibers of the POL; POLm, middle fibers of the POL; POLp, posterior fibers of the POL.

At 0° of flexion, the mean lengths of the anterior, middle, and posterior fibers of the POL were 40.7 ± 4.5 mm, 41.4 ± 5.4 mm, and 42.0 ± 4.4 mm, respectively. With knee flexion between 0° and 120°, all fibers regions of the POL shortened progressively: anterior fibers, –29.2% ± 6.7% (*P* < .001); middle fibers, –42.2% ± 6.5% (*P* < .001); and posterior fibers, –53.9% ± 11.6% (*P* < .001) (Figure 5). The POL was nonisometric (TSR: 29.16% ± 6.74% to 53.92% ± 11.63%; *P* < .001) (Table 2).

### Effect of a Slope-Reducing Osteotomy

**Figure 6.**
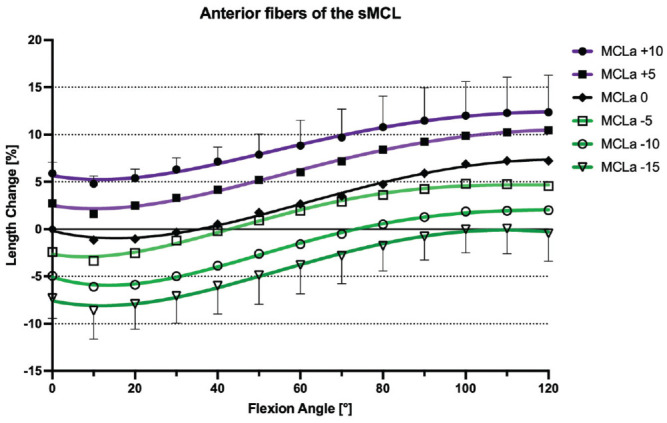
Length change pattern of the anterior fibers of the superficial medial collateral ligament (sMCL) after anterior tibial slope–modifying osteotomy (+10 mm, +5 mm, 0 mm, –5 mm, –10 mm, –15 mm), presented as means ± pooled standard deviations. A slope increase lengthened the anterior fibers, whereas a slope reduction shortened the anterior fibers. Significant length changes are described in the text. MCLa, anterior fibers of the sMCL.

**Figure 7. fig7-03635465241280985:**
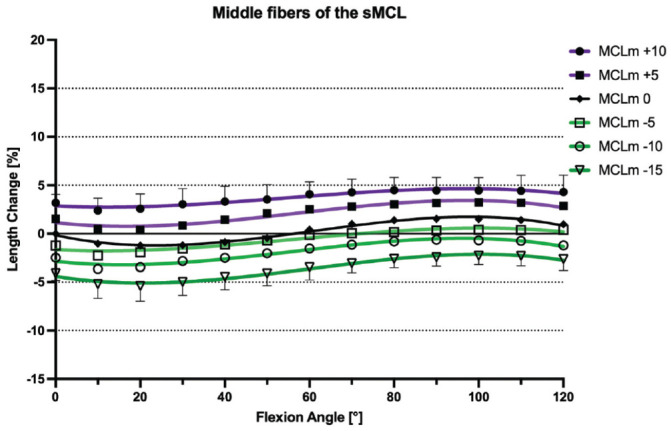
Length change pattern of the middle fibers of the superficial medial collateral ligament (sMCL) after anterior tibial slope–modifying osteotomy (+10 mm, +5 mm, 0 mm, –5 mm, –10 mm, –15 mm), presented as means ± pooled standard deviations. A slope increase tightened the middle fibers, and a slope reduction shortened the middle fibers, while the sine wave behavior was maintained. Significant length changes are described in the text. MCLm, middle fibers of the sMCL.

ACWHTOs of 5 mm, 10 mm, and 15 mm all resulted in significant shortening of the anterior, middle, and posterior sMCL fibers at all angles of knee flexion (*P* < .001). This effect was more pronounced for the anterior fibers compared with the middle and posterior fibers (*P* < .05). With a 15-mm ACWHTO, the anterior sMCL fibers shortened by a mean −6.9% ± 3.0% between 0° and 120° of knee flexion, the middle fibers shortened by −3.9% ± 0.9%, and the posterior fibers by −3.6% ± 2.3% (Figures 6 [Fig fig7-03635465241280985]-8). ACWHTO produced no significant effect on the length change pattern of POL fibers, throughout the range of knee flexion, regardless of the size of the osteotomy (Figure 9).

**Figure 8.**
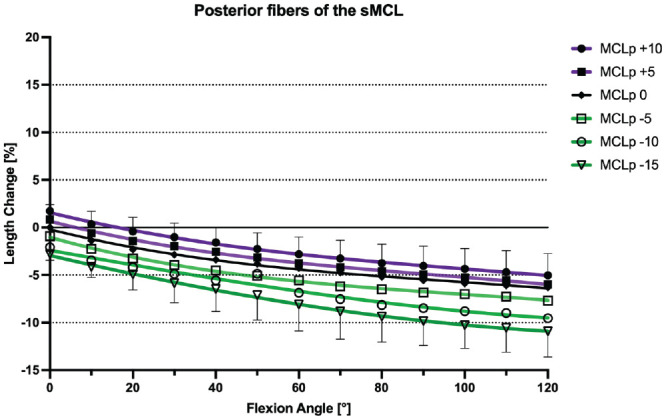
Length change pattern of the posterior fibers of the superficial medial collateral ligament (sMCL) after anterior tibial slope–modifying osteotomy (+10 mm, +5 mm, 0 mm, –5 mm, –10 mm, –15 mm), presented as means ± pooled standard deviations. A slope increase tightened the posterior fibers, whereas a slope reduction shortened the posterior fibers. Significant length changes are described in the text. MCLp, posterior fibers of the sMCL.

**Figure 9.**
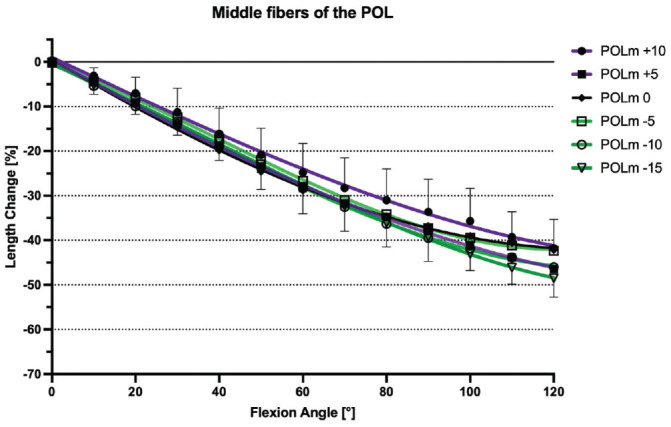
Length change pattern of the middle fibers of the posterior oblique ligament (POL) after tibial slope–modifying osteotomy (+10 mm, +5 mm, 0 mm, –5 mm, –10 mm, –15 mm), presented as means ± pooled standard deviations. Regardless of slope modification, the POL showed no significant change in length throughout the range of knee flexion. POLm, middle fibers of the POL.

### Effect of a Slope-Increasing Osteotomy

A 10-mm AOWHTO resulted in a significant lengthening of the anterior, middle, and posterior sMCL fibers at all angles of knee flexion (*P* < .001). The anterior sMCL fibers lengthened by a mean +5.9% ± 2.3% (*P* < .001), the middle sMCL fibers by 3.5% ± 1.3% (*P* < .05), and the posterior sMCL fibers by 1.6% ± 1.0% (*P* < .05) (Figures 6 [Fig fig7-03635465241280985]-8). A 5-mm AOWHTO lengthened the anterior and middle fibers, but there was no significant increase in the length of the posterior sMCL fibers. AOWHTO produced no effect on the POL length change behavior (Figure 9).

## Discussion

The most important finding of the present study was that a tibial slope–modifying osteotomy changed the length change pattern of the sMCL corresponding to the size of the osteotomy gap. A 15-mm ACWHTO resulted in a mean reduction in the length of the anterior fibers of the sMCL of 6.9%, whereas a 10-mm AOWHTO resulted in a mean increase in length of 5.9%. This effect was more pronounced for the anterior sMCL fibers compared with the posterior fibers, and there was no effect on the length change pattern of the POL fibers. This effect occurs because the osteotomy gap lies between the tibial and femoral attachments of the sMCL and becomes smaller toward its hinge at the posterior tibial cortex adjacent to the posterior cruciate ligament attachment.

The length change patterns of the native MCL have been excessively investigated in several biomechanical studies to understand the complex interaction and recruitment of different fiber regions in restraining valgus and internal/external rotatory loads throughout knee flexion.^6,15,28,48,54^ Consistent with recent studies, we found that the different fiber regions of the sMCL exhibited a reciprocal length change behavior depending on their relation to the medial femoral epicondyle.^28,54^ Fibers attaching posterior to the medial epicondyle lengthened with knee extension, while fibers attaching anterior to the medial epicondyle lengthened with increasing knee flexion. All POL fibers shortened with knee flexion because of their posterior femoral attachment. However, previous studies have not investigated the effect of anterior tibial slope–modifying osteotomies on this length change behavior.

Tibial slope–modifying osteotomy is becoming increasingly popular as an adjunct to the treatment of anterior or posterior knee instabilities.^3,12,44,45^ In 2010, a biomechanical study by Martineau et al^33^ investigated the effect of AOWHTO on MCL strain at 15° of knee flexion using a microstrain transducer. They found no significant change in MCL strain after 5-mm and 10-mm AOWHTOs. In comparison, we found that at 10° of knee flexion, a 5-mm AOWHTO produced a 1.6% increase in strain in the middle fibers of the sMCL and a 10-mm AOWHTO resulted in a 3.2% increase in strain. These differences may result from the different loading protocol (unloaded vs loaded in the present study), test setup (custom-made loading rig vs open-chain extension rig in the present study), and measuring device (microstrain transducer vs optical rotary incremental encoder in the present study).

Our finding that ACWHTO caused a decrease in length of the anterior and middle fibers of the sMCL may have clinical relevance. Considering the mean sMCL length of 90 to 110 mm and the 6.9% and 3.9% shortening of the anterior and middle sMCL fibers after a 15-mm ACWHTO, extensive slope reduction may cause a grade 2 valgus instability according to the Hughston classification.^24,29,32,40^ One may argue that the proximal tibial sMCL attachment may prevent this medial instability, as it is proximal to the osteotomy plane. However, a recent biomechanical study has shown that the proximal tibial sMCL attachment contributes only slightly to the valgus stability of the knee joint.^52^ Furthermore, these fibers are important in the restraint of anteromedial rotatory instability.^22^ Although ACWHTO may be recommended for ACL graft failure to reduce loads on the subsequent ACL grafts, the slackening of anterior fibers of the sMCL may promote anteromedial rotatory instability.^3,10,12,45^ This might have a deleterious effect leading to increased ACL loads and possibly lowering the protective effect of a slope reduction.^7,8^ AOWHTO has the opposite effect, which could potentially lead to overconstraint of the medial compartment and increase the forces on the articular cartilage.^2,43^

The present study has several limitations. Cadaveric knee specimens of older age (mean age, 74 ± 10.3 years) were used, which might not necessarily reflect the bone and soft tissue quality of patients operated on with a slope osteotomy. Braided composite threads running between pins at the tibial and femoral attachment sites were used to simulate the fiber regions of the sMCL. These may not precisely replicate the behavior of the native sMCL during knee flexion and demonstrate subtly different length changes.^15^ However, this methodology has been used previously to simulate sMCL and POL length change patterns.^28^ According to our clinical routine, an infratubercle slope reduction osteotomy was performed, so that the effect of a trans- or supratubercle osteotomy on sMCL and POL length changes cannot be predicted. Furthermore, the initial native slope was not measured and the tibial slope modifications we performed were measured in millimeters of wedge height and not in degrees of tibial slope, potentially leading to different slope corrections depending on the size of the knee. Nonetheless, radiographic slope measurements show moderate inter- and intrarater reliability, so that a 1° to 1-mm transfer of slope modification to wedge height, and thus to the length change of the MCL, may not be possible.^36,55^ The test setup only allowed for the measurement of length changes, whereas actual load and thus the maximum unloaded length could not be assessed.^30^ Furthermore, the potential valgus or anteromedial rotatory instability has not been investigated, so that the clinical consequences are unknown.

## Conclusion

Tibial slope–modifying osteotomies changed the length change pattern of the sMCL such that an AOWHTO increased whereas an ACWHTO decreased the sMCL strain. This effect was most pronounced for the anterior fibers of the sMCL. The length change pattern of the POL remained unaffected by slope-modifying osteotomy. Surgeons should be aware that anterior tibial slope–modifying osteotomies affect the biomechanics of the sMCL. After an extensive ACWHTO, patients may develop a medial or anteromedial instability, while an AOWHTO may overconstrain the medial compartment.
